# Supplementary material for: Modulation of Chitosan-TPP Nanoparticle Properties for Plasmid DNA Vaccines Delivery
Source: Polymers (Basel). 2022 Apr 1;14(7):1443. doi: 10.3390/polym14071443 (PMC9003200; doi:10.3390/polym14071443)
Supplement: Supplementary file 1 [file polymers-14-01443-s001.zip › polymers-1581788-supplementary.pdf]

polymers

Supplementary Material: Modulation of chitosan-TPP nanoparticle properties for plasmid DNA vaccines delivery

Renato Nunes, Ana Sofia Serra, Aiva Simaite, and Ângela Sousa

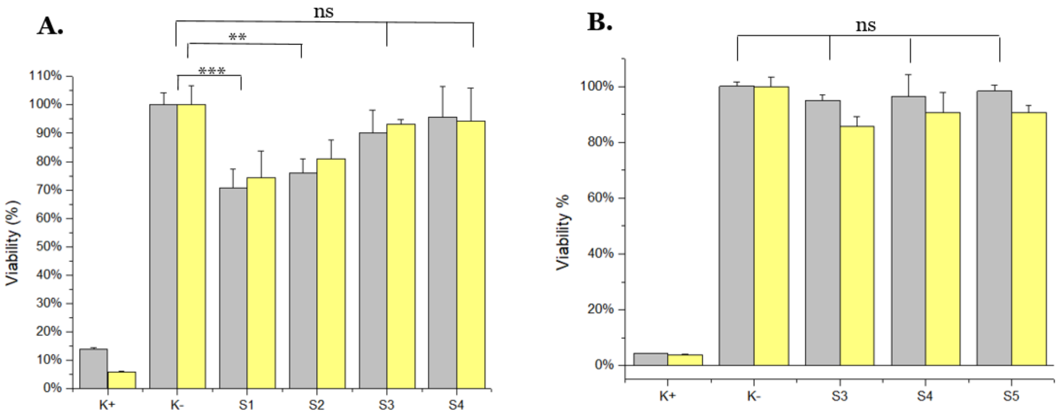

Figure S1. Cell viability after 24 h (gray) and 48 h (yellow) of transfection with CS-TPP-pDNA polyplexes, for hFIB cells (**A**) and for RAW cells (**B**). S1 – NPs centrifuged at 2000 rpm, DNA concentration of 20 µg/mL; S2 - NPs centrifuged at 4000 rpm, DNA concentration of 20 µg/mL; S3 - NPs centrifuged at 6000 rpm, DNA concentration of 20 µg/mL. S4 - NPs centrifuged at 10000 rpm, 10 min, DNA concentration of 20 µg/mL; S5 - NPs centrifuged at 10000 rpm, DNA concentration of 60 µg/mL. Non-transfected cells were used as negative control (K-) and ethanol treated cells were used as positive control (K+) for cytotoxicity. Statistical analysis was made using "one-way ANOVA" with data obtained from three independent measurements (mean ± SD, $n = 3$). The asterisks symbol represents statistical significance (** $p \leq 0.01$; *** $p \leq 0.001$), ns – not significant).
